# Evaluating range-expansion models for calculating nonnative species' expansion rate

**DOI:** 10.1002/ece3.1106

**Published:** 2014-06-30

**Authors:** Sonja Preuss, Matthew Low, Anna Cassel-Lundhagen, Åsa Berggren

**Affiliations:** Department of Ecology, Swedish University of Agricultural SciencesBox 7044, SE-75007, Uppsala, Sweden

**Keywords:** Biological invasion, citizen science, citizen-reported data, dispersal, distribution, insects, invasive species, *Metrioptera roeseli*, orthoptera, range shift

## Abstract

Species range shifts associated with environmental change or biological invasions are increasingly important study areas. However, quantifying range expansion rates may be heavily influenced by methodology and/or sampling bias. We compared expansion rate estimates of Roesel's bush-cricket (*Metrioptera roeselii*, Hagenbach 1822), a nonnative species currently expanding its range in south-central Sweden, from range statistic models based on distance measures (mean, median, 95^th^ gamma quantile, marginal mean, maximum, and conditional maximum) and an area-based method (grid occupancy). We used sampling simulations to determine the sensitivity of the different methods to incomplete sampling across the species' range. For periods when we had comprehensive survey data, range expansion estimates clustered into two groups: (1) those calculated from range margin statistics (gamma, marginal mean, maximum, and conditional maximum: ˜3 km/year), and (2) those calculated from the central tendency (mean and median) and the area-based method of grid occupancy (˜1.5 km/year). Range statistic measures differed greatly in their sensitivity to sampling effort; the proportion of sampling required to achieve an estimate within 10% of the true value ranged from 0.17 to 0.9. Grid occupancy and median were most sensitive to sampling effort, and the maximum and gamma quantile the least. If periods with incomplete sampling were included in the range expansion calculations, this generally lowered the estimates (range 16–72%), with exception of the gamma quantile that was slightly higher (6%). Care should be taken when interpreting rate expansion estimates from data sampled from only a fraction of the full distribution. Methods based on the central tendency will give rates approximately half that of methods based on the range margin. The gamma quantile method appears to be the most robust to incomplete sampling bias and should be considered as the method of choice when sampling the entire distribution is not possible.

## Introduction

Although understanding the factors determining distributions of species in equilibrium with environmental conditions is central to ecology (Andrewartha and Birch 1954; Brown et al. 1996), focus has more recently turned to organisms undergoing range shifts associated with climate change (Parmesan and Yohe 2003; Brooker et al. 2007) and the filling of empty ecological niches during biological invasions (Elith et al. 2010; Václavík and Meentemeyer 2012). Accurate descriptons of range shifts are an important component for predicting future trends; thus, accurate assessment of current and potential distributions of species expanding their current range is a critical step in evaluating environmental impacts and management control options (Drury and Rothlisberger 2008; Keller et al. 2008; Hassall and Thompson 2010). There are many ways to calculate species' range expansions or shifts; some of these methods are complex and require detailed ecological life-history information (e.g., Van den Bosch et al. 1990; Lensink 1997; Hill et al. 2001). However, because detailed ecological knowledge for many species is missing, less complex methods based on species presence data are often used to assess distributional changes.

Species occupancy data collected over large areas and for multiple years can be obtained from a number of sources (e.g., national record data bases, species atlases, surveys, and monitoring programs). Data on species distributions collected by the public and stored in national data bases are generally underused in research and management (Goffredo et al. 2010), although being valuable for estimating changes in species distributions (Snäll et al. 2011). Methods using occupancy data in range expansion assessment can be crudely categorized as those that are area based and those that are distance based. In area-based methods, changes in range size are quantified by measuring the occupied area (counting the number of occupied grid cells (Ward 2005), with the rate of change calculated from the increase of occupied grids over time (Hill et al. 2001). In distance-based methods, range shifts are assessed by measuring the geographical distances between observations from different time periods with the first observation of the species in a specific location; including the mean, median, maximum, or marginal mean (mean of the ten most distant observations) of the annual distances to calculate the expansion rate of the species (Hassall and Thompson 2010).

**Figure 1:**
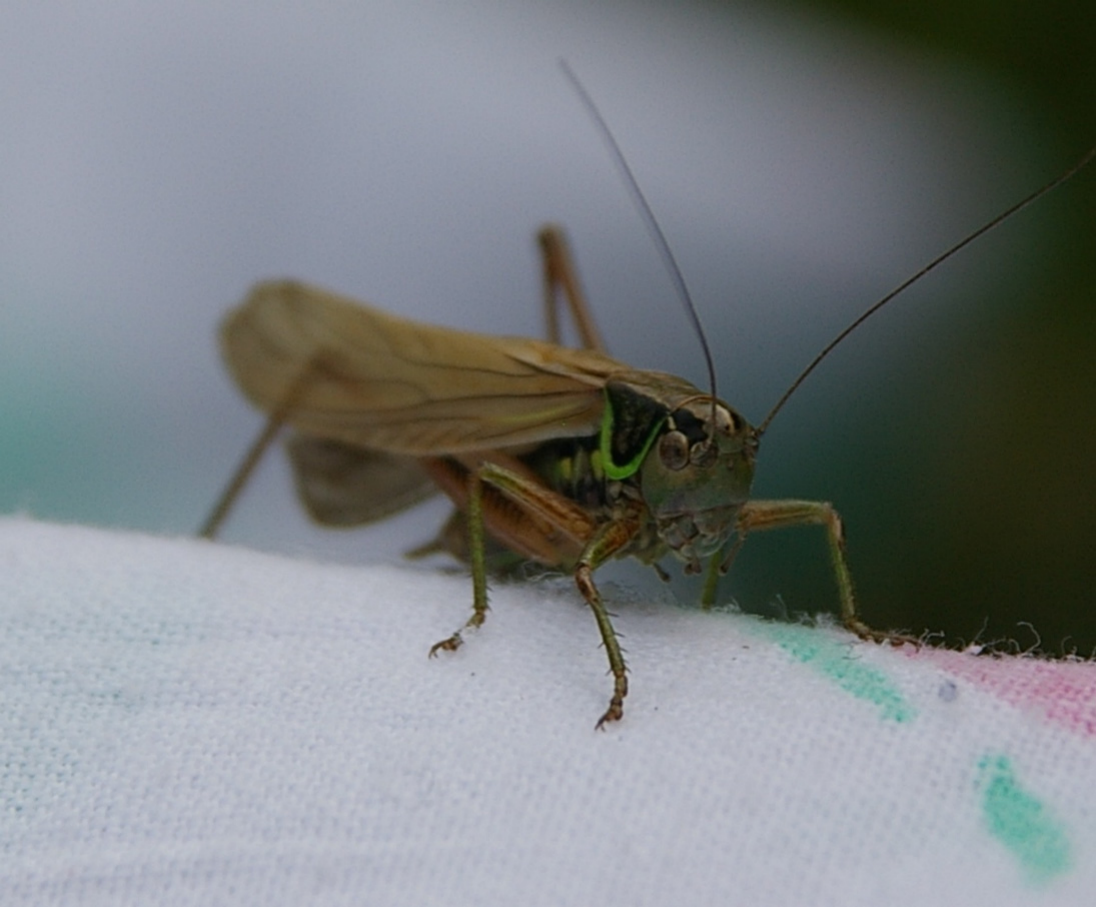
A male Roesel's bush-cricket (*Metrioptera roeselii*). This is a macropterous (long-winged) morph thought to be important for longer distance dispersal.

Despite various methods being used independently in different studies to calculate range expansion, an evaluation of their comparative performance and sensitivity to sampling effort, that is, number of species records needed for an accurate assessment of range expansion rate, is generally missing (but see Hassall and Thompson 2010). Thus, the main aim of our study was to compare the performance of seven widely used range-expansion models to quantify the rate of range expansion and the sensitivity to sampling effort in a Swedish population of the Roesel's bush-cricket (*Metrioptera roeselii*; Fig. 1). This orthopteran is nonnative to Sweden and currently expanding in its range, not only in Sweden but also in other European countries (Pettersson 1996; Simmons and Thomas 2004; Gardiner 2009; Hochkirch and Damerau 2009; Species Gateway 2010). The Swedish population of Roesel's bush-cricket is ideal for evaluating range expansion models, because the species is easy to record in the field, there are long-term records in presence-based data bases and the population has been the subject of two large-scale censuses in 1989–1990 and 2008–2010. These existing data make it possible to estimate expansion rate of the species using different commonly used methods and compare model predictions and performance. For this, we used the initial record and the two large-scale survey data on the distribution of *M. roeselii* in central Sweden to: (1) calculate the species' expansion rate using different range-expansion models, to compare the estimates obtained from each method, and (2) evaluate how robust the different distance-based methods are to sampling effort (range 1–100%) through simulation. We then used these sampling simulation results to help interpret changes in range expansion estimates when we recalculated expansion rates for each model using summary data for all years where records exist, which included incidental observations recorded in the Species Gateway (i.e., data with potential sampling bias). Thus, our aim was not primarily to document the ‘true’ rate of expansion of this species, but rather to highlight the characteristics and limitations of commonly used range-expansion models under conditions of incomplete sampling effort.

## Methods

### Model species

*Metrioptera roeselii* (Orthoptera: Tettigoniidae, Hagenbach 1822; Fig. 1) is a small (12–18 mm) bush-cricket commonly found in grasslands of central and northern Europe (Bellmann 2006). In Sweden, *M. roeselii* occurs predominantly in the Lake Mälaren region and both the position of the population core area and population genetic data strongly suggest that the species was introduced here via sea cargo (de Jong and Kindvall 1991; Kaňuch et al. 2013). *Metrioptera roeselii* is an omnivorous generalist that prefers tall grassland habitats (Marshall and Haes 1988). Detailed studies on the ecology of the species (e.g., Ingrisch 1984; Poniatowski and Fartmann 2005; Holzhauer et al. 2006; Berggren 2009) and movement behavior (Berggren et al. 2002; Berggren 2004, 2005; Poniatowski and Fartmann 2010) have increased the understanding of how *M. roeselii* responds to local biotic and abiotic factors. Its presence in the agricultural landscape can be predicted by the amount of arable land, which is closely associated with grassy field margins and ditches (Berggren et al. 2001; Preuss et al. 2011). The majority of this wing polymorphic species are short-winged and usually disperse short distances by walking and jumping (Berggren et al. 2001). High population density and favorable weather conditions can trigger the development of long-winged morphs that are capable of flight dispersal of up to 19 km (Hochkirch and Damerau 2009).

### Large-scale species occupancy data

**Figure 2:**
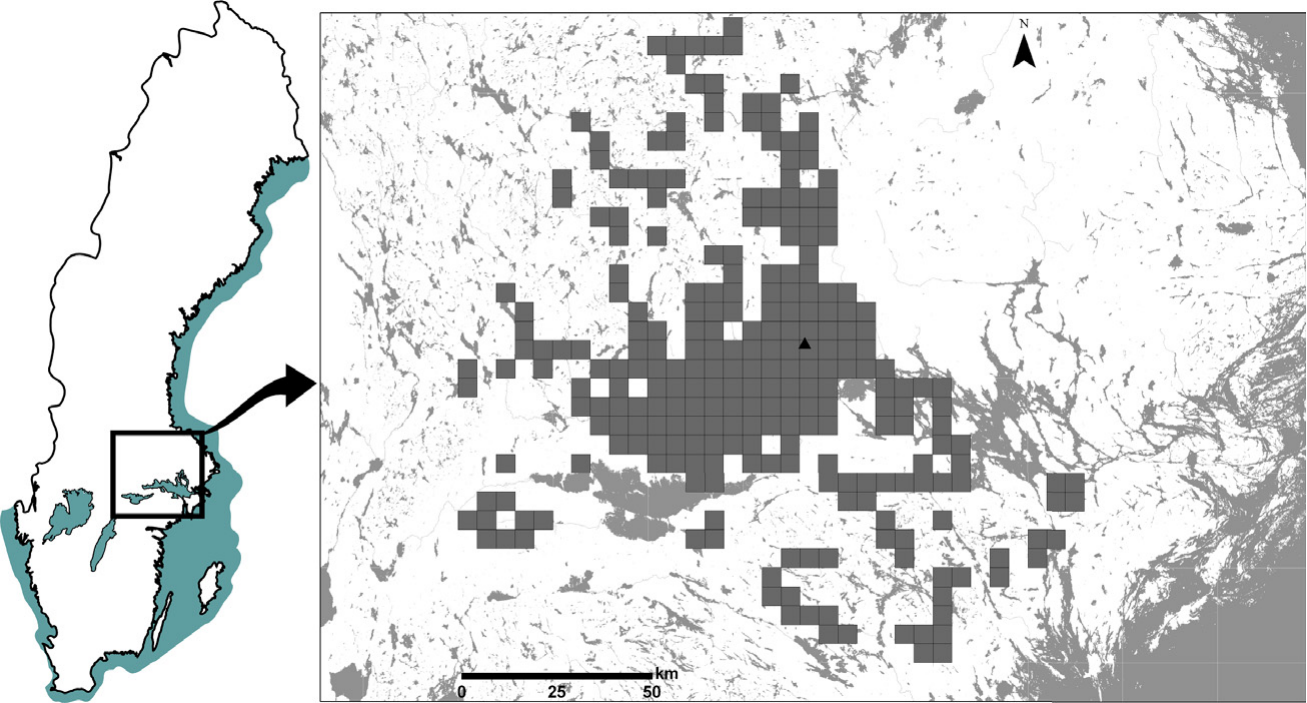
Geographical distribution of presence data in 5 × 5 km grid squares for *Metrioptera roeselii* (midpoint: Lat. 59°44′N, Long. 16°52′E) in south-central Sweden (*n* = 366). Data are gathered from two comprehensive surveys (1989–1990, 2008–2010) and from the national record data base – Species Gateway (1981–2010).

**Figure 3:**
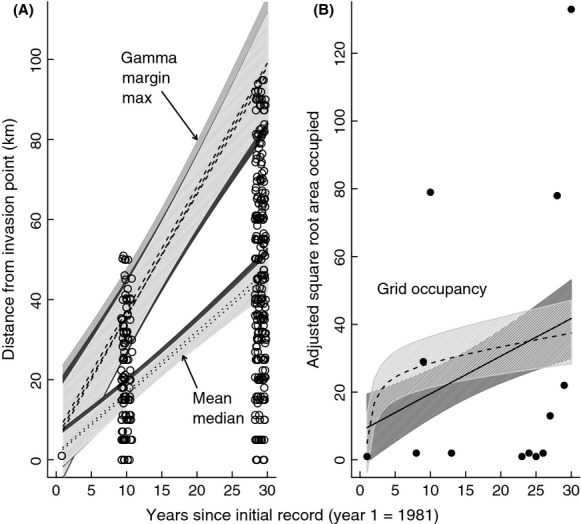
Model predictions (± SE) showing the rate of range expansion (slope of the prediction line = km/year) for different model types from 1981 to 2010. In panel (A), dashed lines represent distance-based methods at the range margin (gamma, marginal mean, and maximum), and dotted lines represent methods using the central tendency (mean and median). Open circles show the location of squares relative to the invasion point (km) for the main survey periods. In panel (B) grid occupancy models show linear (solid line) and nonlinear (dashed line) predictions adjusted (/√*π*) so the slope of the line is the rate of range expansion. Closed circles show the total number of grid squares surveyed during each year.

In order to assess the rate of range expansion of *M. roeselii* in central Sweden since the first record in 1981, we combined all available data on this species' distribution (1981–2010) from the national record data base (Species Gateway http://www.artportalen.se, 510 observations) and large-scale surveys that were carried out in 1989–1990 and 2008–2010 (de Jong and Kindvall 1991; Preuss et al. 2011) (Figs. 2, 3). The Species Gateway is a species data base administered by the Swedish Species Information Center (ArtDatabanken), to which the general public, scientists, organizations, and authorities can report species observations. There is increasing interest to report species in Sweden via the Species Gateway; in the beginning of 2012, there were more than 32 million observations across all species. The observations include data on geographical position, abundance and in some cases data on life-history stage. All reports are subsequently verified by taxonomic specialists. The large-scale surveys on the distribution of *M. roeselii* in central Sweden were conducted during in 1989–1990 and 2008–2010, centered on the Lake Mälaren region (midpoint 59°44′N, 16°52′E) where the species was originally introduced. Known locations of *M. roeselii* (de Jong and Kindvall 1991; Berggren et al. 2001; Species Gateway 2010) were used as starting points for the surveys to map the distribution of the species. Based on an established method (de Jong and Kindvall 1991; Berggren et al. 2001), cars were used to conduct auditory surveys on sunny days, between 10 am–5 pm, from mid-July until the end of August. Because the species' call is loud and distinctive and can be heard for distances of >10 m (Fischer et al. 1997; Bellmann 2006), it is possible to listen for stridulating males from the car window while driving slowly (20–30 km/h) along countryside roads. Because the bush-cricket is generally restricted to agricultural areas and grasslands, and access to these areas is possible on public and farm roads, this ensured most potential sites were surveyed. When detected, the identity of *M. roeselii* was always confirmed by stopping the car and surveying the local area on foot; in almost all instances, multiple males were heard stridulating in the area suggesting an established (or establishing) local population. Survey routes and observations of *M. roeselii* were noted on maps (1989–1990) and by using a GPS (2008–2010, Garmin 60XL).

Because the 1989–1990 survey data were only available at a 5 × 5 km grid resolution, and models for quantifying the rate of range expansion use presence data in grid format, we converted the point location data from the national record data base and the 2008–2010 survey to 5 × 5 km grid data comparable to the 1989–1990 survey data (cf. Hill et al. 2001). This provided data from 366 different occupied grid squares during 14 years between 1981 and 2010, covering an area of 9150 km^2^ (Figs. 2, 3).

### Methods assessing the rate of range expansion

We compared seven different models that have been previously used to calculate expansion rates of species with grid-based occupancy data (butterflies: Hill et al. 2001; Pöyry et al. 2009; dragonflies: Hassall and Thompson 2010; marine macrophytes: Mineur et al. 2010). These models traditionally use a linear regression in which the area or distance measures of range size or range shift are plotted against time (year of the observations), with the slope of the regression being used to calculate range expansion speed (e.g., km/year). In our study, we chose the geographical position of the first record in the national data base (i.e., 1981; Species Gateway 2010) as the reference point for measuring distances to occupied grid cells in the subsequent years. We are confident that this position closely reflects the invasion origin because surveys of the surrounding area at the time (1981) did not locate other populations, and the grid square is on the shore of lake Mälaren which is the most likely point of entry of *M. roeselii* to Sweden (de Jong and Kindvall 1991); subsequent genetic studies strongly suggest that this is (or close to) the point of origin (Kaňuch et al. 2013).

The seven range-expansion models were as follows: (1) grid occupancy (Hill et al. 2001), where the number of occupied grid cells (i.e., the square root of the occupied area) is used to estimate changes in the range size over time; (2) mean distance (Hassall and Thompson 2010), where the mean from the initial location record to all occupied grid cells of the observation year is calculated; (3) median distance (Hassall and Thompson 2010), where the annual median distance from the first location record is used; (4) gamma quantile (Hassall and Thompson 2010), where a gamma distribution is fitted to the annual distance data between occupied grid cells and the first location record; the gamma distribution is a positive continuous distribution and is thus well suited to modeling positive continuous range expansion data, with the 95^th^ quantile of the distribution used as the measure of the position of the species range margin in a given year; (5) marginal mean (Pöyry et al. 2009; Hassall and Thompson 2010), where the mean of the ten outermost occupied grid cells is used to describe the location of the range margin; (6) maximum distance (Hassall and Thompson 2010), which measures the range margin as the distance between the first record to the most distant occupied grid cell per year; (7) conditional maximum (Mineur et al. 2010), which uses the same principle as the maximum, but only allows values to increase over time (i.e., if a maximum value is less than previous years, the previous year's value is retained as the maximum). In all cases except the grid occupancy model (an area-based method), the rate of range expansion is the slope of the regression (Ward 2005). For grid occupancy, the marginal velocity of range expansion is calculated by dividing the slope of the regression by the square root of pi (Lensink 1997; Hill et al. 2001).

To compare range-expansion rate estimates for the different models, we initially restricted our data to the three survey periods (1981; 1989–1990; 2008–2010) where data were pooled for each period, thus reflecting the initial record and surveys at years 10 and 30. This was to ensure accurate estimates for comparison, since subsampling the occupied range is likely to result in biased range metrics (see below). Because expansion rates may not be linear over time (Shigesada and Kawasaki 1997), for each of the seven range-expansion models we compared three different model forms relative to year since detection (*t*): (1) a simple linear model described by an intercept and slope [*a* + *b* × *t*]; (2) a cyrtoid functional response model [*a* + *t*/*b* × *t*]; and (3) an exponential growth model [*a* × *e*^b*t*^]. We used the ‘nls’ function in R (R Development Core Team 2011) to fit models and compare model sets using AIC_c_ (Burnham and Anderson 2002). Depending on which model received the most support, we then used 20000 iterations of a Gibb's MCMC sampler (JAGS; Plummer 2003) to generate the 95% confidence (credible) intervals around the range expansion estimates. We used this approach for two reasons; first, it allowed us to generate an estimate of the range expansion rate (with CIs) for nonlinear functions by sampling from the posterior distribution of the derivative (i.e., slope) of the function. Second, it allowed us to compare range expansion estimates between models (e.g., median versus gamma) and directly calculate the probability that the estimates differed from each other.

### Effect of sampling effort on range expansion calculations

We were interested in determining if data from outside the survey periods (e.g., incidental observations) with much lower sampling effort would bias our range expansion estimates. In contrast to the three comprehensive surveys of the species range in 1981, 1989–1990, and 2008–2010, citizen-reported data obtained from the national record data base were associated with a lower sampling effort, covering only a fraction of the occupied range at different times (sometimes only a single record). Because estimates of range expansion rates are potentially susceptible to bias if only a proportion of occupied sites are sampled (Hassall and Thompson 2010), we quantified this bias for our data and each range-expansion statistic by using a random subsampling approach from years for which we had accurate *M. roeselii* surveys.

For this, we created a function in the statistical programming language R (R Development Core Team 2011) to randomly subsample from 1 to 100% the presence data from the latest pooled survey period 2008–2010 (*N* = 233 occupied grid cells) to test for the effect of sampling effort, that is, number of annual records used for the calculation of the expansion rate. Because the rate of range expansion in the grid occupancy model is estimated from the absolute number of grid cells occupied, any subsample will give a downward-biased estimate proportional to the degree of subsampling and so it was calculated directly. From each subsample, the range statistics of the mean, median, gamma quantile, marginal mean, and maximum were calculated. This was repeated 10,000 times for each level of sampling effort (1–100%) to generate a distribution for each range statistic at each sampling level. From this, we calculated the 95% confidence intervals (CIs) for the different range statistics at each level of sampling effort. From these data, we could calculate the minimum sampling proportion required to ensure that a range statistic calculated from a subsample did not deviate more than 5 or 10% from the true value (i.e., the true value lay within the 95% CIs of the value estimated from a subsample) to obtain an accurate estimate of the rate of range expansion (cf. Hassall and Thompson 2010).

Based on these thresholds, it was obvious that yearly range statistics calculated from data collected outside the main survey periods were likely to be strongly biased, potentially influencing range expansion calculations. To investigate how incomplete and irregular levels of data collection effort may influence range expansion estimates, we recalculated the expansion rates of the mean, median, gamma, marginal mean, maximum, and conditional maximum models using range statistic data calculated for each of the 14 years where we had records. This meant that the survey periods were split into their yearly values (i.e., rather than pooling the 2008–2010 survey into one comprehensive survey of the region, it was divided into the three component years). Similarly, years with only a few or a single citizen-reported data point were included where possible (e.g., for a single point mean, median, and maximum are possible, but not gamma because estimating a gamma distribution requires at least 2 data points).

## Results

### Range expansion estimates using comprehensive survey data

For all distance-based methods (mean, median, gamma, marginal mean, and maximum), the simple linear model always had greater support (i.e., lower AIC_c_) than fitting a nonlinear function (Fig. 3A). For the area-based method (grid occupancy), the cyrtoid function had twice the support as the linear function (ΔAIC_c_ = 1.6); thus, we calculated the estimated expansion rate from both the linear and nonlinear functions for grid occupancy (Table 1; Fig. 3B).

**Table 1:** Estimated rates of range expansion (km/year) derived from different range expansion models. The estimates using survey data are based only on the three periods when the area around the invasion point was comprehensively surveyed to the range margin (1981; 1989–1990; 2008–2010), and include 95% CIs in parentheses. Sampling accuracy shows the proportion of occupied sites (i.e., 5 × 5 km grid squares) that need to be sampled to be confident that the range statistic (e.g., mean) is within 5 (or 10)% of the true value. The estimates using yearly data include all years where records exist (1981–2012), regardless of how large an area was surveyed in that year; thus these estimates include years with highly biased data

Range expansion model	Estimate [km/year] using survey data (95% CIs)	Sampling accuracy within 5 (or 10)%	Estimate [km/year] using yearly data
Grid occupancy (linear)	1.11 (0.55–1.66)	0.95 (0.90)	0.31
Grid occupancy (nonlinear)	1.58 (1.02–2.21)	0.95 (0.90)	0.69
Mean	1.52 (0.97–2.07)	0.66 (0.33)	1.28
Median	1.50 (1.05–1.95)	0.97 (0.83)	1.24
Gamma	3.03 (1.62–4.45)	0.51 (0.20)	3.21
Marginal mean	3.12 (1.85–4.41)	0.58 (0.38)	1.97
Maximum	3.09 (1.49–4.69)	0.36 (0.17)	2.59
Conditional Maximum^1^	3.09 (1.49–4.69)	–	1.45

1 Sampling accuracy is not given for conditional maximum because its value is conditional on previous years' values that are not included in the data simulation.

There were two distinct groups of range expansion estimates, with distance-based methods calculated at the range margin (gamma, marginal mean, maximum, and conditional maximum) all giving very similar results (∼3 km/year; Table 1; Fig. 3A). The second grouping was for distance-based methods calculated from the central tendency (mean and median) and the area-based method of grid occupancy, with these being roughly half those calculated from the range margin (∼1.5 km/year; Table 1; Fig. 3). By conducting pair-wise comparisons of the posterior distributions of these estimates, there was a 96% probability (range 93–99%) that the range margin group had higher range expansion estimates than the central tendency group (using Bayesian derived quantities from the subtraction of one estimate from another and seeing the proportion of the resulting distribution that overlapped zero).

### Effect of sampling effort on range expansion calculations

**Figure 4:**
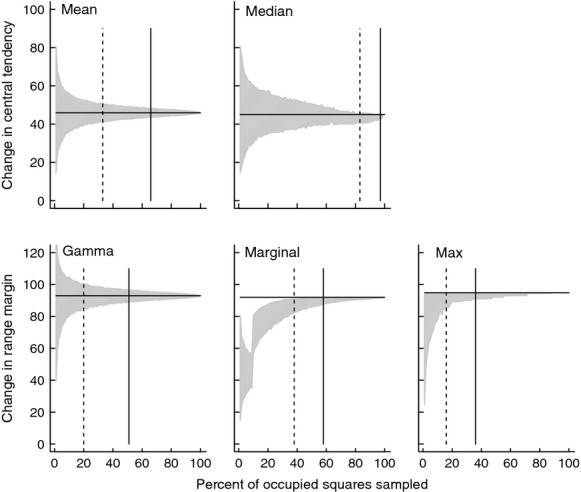
Sensitivity of five range expansion statistics (mean, median, gamma quantile, marginal mean, and maximum) to sampling effort ranging from 1 to 100% of the 2008–2010 survey data. The true state of the system is the horizontal line, calculated when 100% of the data are sampled. At each level of sampling effort, the gray area shows the 95% confidence interval (i.e., 0.025 & 0.975 quantiles) of 10,000 resamples. The vertical solid line indicates the minimum level of sampling effort required for the 95% CIs to be within 5% of the true value; the vertical dashed line is within 10% of the true value.

Range expansion models differed in their sensitivity to sampling effort when the number of records used to calculate the range statistic was varied from 1 to 100% of the total records (Fig. 4). The variation in sensitivity was remarkably large with the proportion of sampling required to get an estimate within 10% of the true value ranging from 0.17 to 0.9, and to be within 5% the range was 0.36–0.95 (Table 1). The methods most sensitive to sampling effort were the grid occupancy and median models, and the least sensitive were the maximum and gamma models (Table 1; Fig. 4). For the mean, median, and gamma models, reduced sampling produced both under- and overestimates of the true value, while the maximum, marginal mean, and grid occupancy always produce an underestimate (Fig. 4).

The effect of using yearly summary data, regardless of sampling effort, to calculate range expansion estimates can be seen in the right hand column of Table 1. These estimates were generally lower (range 16–72%) than those derived from the survey data; the one exception being the gamma quantile model that was slightly higher (∼6%; Table 1). The models with the greatest change in point estimates (36–72% lower) were the grid occupancy, conditional maximum, and marginal mean; based on comparisons of their posterior distributions there was a > 90% probability that the estimates from the ‘biased’ models were lower than those using only the survey data. Those with the smallest change (6–17%) were the gamma, maximum, mean, and median (Table 1), and there was little evidence that these differences represented any real change (probability of difference <70%).

## Discussion

Estimates of range expansion rates using the change in a range statistic measure over time are a function of two key modeling components: calculation of the yearly range statistic from the distribution data, and the fitting of a model to quantify the temporal trend across years. Range statistics can be calculated from the observed area occupied (grid occupancy), from the central tendency of the distribution of observations (mean and median) or from the range margin of the observed distribution (95^th^ gamma quantile, marginal mean, maximum, and conditional maximum). Because range statistics have their own mathematical properties, not only may they influence the calculation of range expansion rates in specific ways but incomplete sampling may also affect them differently (Hassall and Thompson 2010). Thus, when interpreting range expansion estimates, these factors need to be considered in addition to the type of model fit used to explain temporal trends (e.g., linear versus nonlinear; Shigesada and Kawasaki 1997). We discuss these issues and the implications for citizen-collected data below.

### Range statistics and range expansion models

The analysis of distribution data collected between 1981 and 2010 estimated that *M. roeselii* had been expanding its range in central Sweden at a rate between 1.11 to 3.12 km/year depending on the model type used (Table 1). Despite there being relatively large variation in these range expansion estimates, much of it was predictable based on the mathematical properties of the range statistics used. For the distance-based methods, rates calculated from the range margin were roughly double those calculated from the central tendency (∼3 vs. ∼1.5 km/year, respectively); in general the central tendency of a group of values will generally increase at half the rate of the maximum (all else being equal; see Fig. 3A). However, this need not always be the case because long-distance dispersers at the range margin are likely to comprise a disproportionately small proportion of the population (and thus have a relatively small influence on the mean and median), despite having potentially large effects on range margin statistics. The establishment of pioneer populations is often the main factor driving rapid increases in the occupied area (Kovacs et al. 2011), and may be one reason why models using range changes at the distribution margin in other Orthopterans (e.g., *Conocephalus discolor*), can be up to six times larger compared to those at the core of the range (Simmons 2003). Range expansion estimates using the median might be expected to be lower than the mean because dispersal distance data are often positively skewed, with the majority of individuals dispersing short distances and few individuals dispersing far (Preuss 2012). In such cases, central tendency models may be less well suited to describing a dispersal pattern created by two different dispersal behaviors: one slow and continuous dispersal and another infrequent long-distance dispersal.

The grid occupancy model uses average radial distance (i.e., square root of the occupied area divided by the square root of pi) and thus should give results comparable to other distance-based methods at the range margin (in this study ∼3 km/year). However, the linear form predicted the lowest rate of range expansion (1.11 km/year) and the nonlinear form was comparable to the central tendency models (∼1.5 km/year). It is important to note that the grid occupancy model assumes dispersal according to a simple diffusion model (Van den Bosch et al. 1990; Lensink 1997) with the range expanding in approximately concentric circles that are largely occupied, even if the expansion front is irregular (Shigesada and Kawasaki 1997). However, if we consider the occupied area of *M. roeselii* in Fig. 2, we see that many squares within the dispersal region are unoccupied; if we assume a 92 km radius based on the gamma quantile, then the proportion of occupied squares is only 0.21. This low rate of occupancy may be because of incomplete detection, habitat avoidance (particularly the large regions of forest in this area; Preuss et al. 2011) or expansion at the periphery occurring through the formation of satellite colonies from long-distance dispersers (Shigesada and Kawasaki 1997). It is likely that the violation of assumptions of this model is, at least partly, responsible for the nonlinear function having a better fit to the data, when it should have been similar to other range margin models that were fit using a simple linear regression. Although range expansion rates across time are likely to be more complex than a simple linear fit would suggest (Shigesada and Kawasaki 1997), because the population is currently undergoing a rapid expansion phase and we had only a limited number of survey points (effectively only three; 1981, 1989–1990 & 2008–2010), a linear fit to the data is not unsurprising (Fig. 3).

### Sensitivity to incomplete sampling

The grid occupancy model (as discussed above) assumes extensive colonization within the ‘circular’ range area; as the number of occupied squares decreases from saturation, the range expansion estimate declines as a function of the square root of the occupied area (e.g., if only one quarter of the area is occupied, the range expansion rate estimate will decline by half). Thus, to obtain reliable range expansion estimates using grid occupancy, extensive sampling across the entire distribution range at regular time intervals is required. For example, in a study on the range dynamics of the hooded warbler (*Setophaga citrine*) estimates of range expansion were highly sensitive to sampling effort and location; increasing sampling time by 100 h and surveying additional squares in the vicinity of occupied squares led to an increase of the estimated expansion rates by 15 and 38%, respectively (Melles et al. 2011). For *M. roeselii* in Sweden, sampling effort was highly variable across all years because grid occupancy data originated from multiple sources (surveys versus incidental observations). When we included data from years in which the species occupied area was largely undersampled, it led to an underestimation of the rate of range expansion in *M. roeselii* by an order of magnitude. Therefore, this method would be most suitable for species where atlas data are available or monitoring programs with the appropriate funding and staff are in place.

Distance-based models showed large variation in their sensitivity to subsampling (Table 1); data from years with low sampling effort can produce extremely uncertain estimates depending on the method used. Previously, studies have used low thresholds without quantifying the sensitivity of this on their estimates: Hickling et al. (2006) had a threshold of 20 records and Hassall and Thompson (2010) analyzing historical distribution data of Odonata calculated that at least 45 records/year are necessary to estimate range expansion with 90% accuracy. Although this suggests previous studies might have underestimated the uncertainties, it does not necessarily mean their estimates are systematically biased. When considering the effect of incomplete sampling there is the uncertainty associated with calculating the range statistic for each time period in the analysis, with this uncertainty declining as a function of sampling effort (Fig. 4). However in addition, there is the degree of bias generated by subsampling; this effect becomes evident when we compare the uncertainty estimates generated for the mean, median and gamma models with the marginal mean, maximum, and grid occupancy. The marginal mean, maximum, and grid occupancy will always be downwardly biased as sampling effort is reduced (with the degree of this bias a function of sampling effort), while this will not generally be the case for models fitting the mean, median and gamma range statistics because they are just as likely to over- as underestimate the true value. This means that if enough years of data are collected, the model fit will bisect these uncertainties and converge on the true range expansion rate (see Hassall and Thompson 2010 for examples of this). Thus, when choosing a method to best estimate range expansion when sampling is incomplete (or the degree of sampling unknown), consideration should be given to methods that are relatively insensitive to sampling in the calculation of the range statistic, and do not give systematic downward biases.

### Implications and recommendations

Based on our results, it appears the 95^th^ gamma quantile is the method of choice; unlike other range margin models it does not give any systematic bias when sampling is reduced, and unlike the central tendency models it is relatively insensitive to incomplete sampling. However, there are specific instances when other range-margin models should be considered, especially when restricted sampling can be focused on the range margin (in our study, we assumed incomplete sampling was randomly assigned across the entire distribution). One practical advantage of measuring range expansion at the range front is that fewer observations are needed from a restricted geographic area to estimate expansion rate. Sensitivity analysis showed that for the maximum, a sampling effort of only 16% of the available records was sufficient to obtain reliable expansion estimates. This estimate of sampling effort was based on the entire distributional area and could conceivably be greatly reduced if surveys were specifically targeted to range margins. However, since estimates would then be derived from only a small number of observations, spatial and temporal aspects will become increasingly important to consider in the sampling strategy. Stratified surveys and repeated sampling of specific locations over time has been found a useful approach in monitoring the range expansion of widespread nonnative plants in the United Kingdom (Hulme 2003). Previous use of diffusion models has shown severe underestimations of expansion rate (e.g., 20 times slower than observed rate in the nonnative cereal leaf beetle *Oulema melanopus*) (Andow et al. 1990). We believe that for nonnative species it is appropriate to adopt a precautionary approach (Hulme 2003), and focus the expansion models on data from the species distribution boundary.

Because organized large-scale surveys at regular time intervals are financially and time-costly, citizen-collected data have been promoted as a solution for estimating species distributions (Gardiner 2009; Snäll et al. 2011); however, a certain level of citizen participation is required to adequately sample the distribution area. One possibility for improving the usefulness of citizen-reported data could be to encourage its collection in areas where satellite populations are establishing at the distribution margin. Because the amount of information required from the species range margin for obtaining accurate estimates of range expansion is relatively small, even restricted information on species presence from these areas can provide useful data for accurate range expansion estimations. In addition, single observations can provide valuable information for directing future survey efforts and management actions as small systematic changes and trends may become important in the longer term (Parmesan and Yohe 2003). With increased citizen effort focused to these margin areas, sufficient amounts of data could be effectively gathered in short periods and over a large spatial extent. This early detection of pioneer populations at the outer range margin is also important for the effective management of invasive organisms (Moody and Mack 1988; Hulme 2003). While it is being increasingly recognized that national data bases with citizen-reported records are an important source of information to assess the ongoing spread of nonnative species (Aslan and Rejmánek 2010), it should be stressed that these sources of information cannot always replace structured and targeted surveys. As our study shows, low sampling effort in years that only included opportunistic observations had potentially large negative effects on range expansion estimates and thus these records cannot always be reliably utilized for high accuracy in range-shift estimations.
